# Promotion of Intestinal Epithelial Cell Turnover by Commensal Bacteria: Role of Short-Chain Fatty Acids

**DOI:** 10.1371/journal.pone.0156334

**Published:** 2016-05-27

**Authors:** Jung-ha Park, Takenori Kotani, Tasuku Konno, Jajar Setiawan, Yasuaki Kitamura, Shinya Imada, Yutaro Usui, Naoya Hatano, Masakazu Shinohara, Yasuyuki Saito, Yoji Murata, Takashi Matozaki

**Affiliations:** 1 Division of Molecular and Cellular Signaling, Department of Biochemistry and Molecular Biology, Kobe University Graduate School of Medicine, Kobe, Japan; 2 The Integrated Center for Mass Spectrometry, Kobe University Graduate School of Medicine, Kobe, Japan; National Cancer Institute, UNITED STATES

## Abstract

The life span of intestinal epithelial cells (IECs) is short (3–5 days), and its regulation is thought to be important for homeostasis of the intestinal epithelium. We have now investigated the role of commensal bacteria in regulation of IEC turnover in the small intestine. The proliferative activity of IECs in intestinal crypts as well as the migration of these cells along the crypt-villus axis were markedly attenuated both in germ-free mice and in specific pathogen–free (SPF) mice treated with a mixture of antibiotics, with antibiotics selective for Gram-positive bacteria being most effective in this regard. Oral administration of chloroform-treated feces of SPF mice to germ-free mice resulted in a marked increase in IEC turnover, suggesting that spore-forming Gram-positive bacteria contribute to this effect. Oral administration of short-chain fatty acids (SCFAs) as bacterial fermentation products also restored the turnover of IECs in antibiotic-treated SPF mice as well as promoted the development of intestinal organoids in vitro. Antibiotic treatment reduced the phosphorylation levels of ERK, ribosomal protein S6, and STAT3 in IECs of SPF mice. Our results thus suggest that Gram-positive commensal bacteria are a major determinant of IEC turnover, and that their stimulatory effect is mediated by SCFAs.

## Introduction

Terminally differentiated and mature cells possess a distinct life span that is strictly regulated in order to achieve and maintain homeostasis and structural integrity in a variety of tissues and organs. The life span of intestinal absorptive enterocytes and of skin keratinocytes, for example, is relatively short (3 to 5 days in mouse for the former, and a few weeks in mouse for the latter), whereas mouse neurons such as Purkinje cells survive for a year [1–5]. The life span of such mature cells is thought to be regulated by both positive and negative survival signaling that is elicited in a cell-autonomous manner or by extracellular stimuli. The specific molecular mechanisms underlying the regulation of life span in many mature cell types remain largely unknown, however.

Intestinal epithelial cells (IECs) of the mammalian small and large intestine are regenerated continuously throughout adulthood from stem cells that reside in a region near the base of intestinal crypts [6, 7]. These stem cells generate proliferating progeny, known as transient amplifying (TA) cells, that migrate out of the stem cell niche, cease to proliferate, and initiate differentiation into the various cell lineages of mature intestinal villi, including absorptive enterocytes, mucin-producing goblet cells, peptide hormone–secreting enteroendocrine cells, and antimicrobial peptide–producing Paneth cells. Cells of the first three of these four lineages mature and migrate up the crypt toward the tip of intestinal villi, whereas Paneth cells migrate down to the base of the crypt. Absorptive enterocytes in particular are released into the gut lumen after they have migrated to the tip of the villi where cell apoptosis and shedding occur. The continuous production of new IECs in each crypt is thus balanced by the elimination of older cells at the luminal side of the intestine, resulting in a rapid turnover of IECs.

Such homeostasis of the intestinal epithelium is thought to be coordinately regulated both by intrinsic elements, such as Wnt ligands and various growth factors, and by extrinsic elements such as gut commensal bacteria [8, 9]. The Wnt signaling pathway plays an important role in the maintenance of leucine-rich repeat–containing G protein–coupled receptor 5 (Lgr5)–positive stem cells as well as in the generation of Paneth cells in the crypt [8]. Indeed, Paneth cells neighboring Lgr5-positive stem cells are thought to contribute to the stem cell niche by secreting Wnt ligands such as Wnt3 [10]. The Wnt signaling pathway is also implicated in positive regulation of TA cell proliferation [11]. In contrast, the Notch pathway is thought to be important for expression of Hes1, which in turn prevents expression of a factor, known as Atoh1 (or Math1), that promotes the development of secretory cells including goblet, enteroendocrine, and Paneth cells [12]. Furthermore, the Ras–ERK (extracellular signal–regulated kinase) signaling pathway has been recently shown to promote the generation of both absorptive enterocytes and goblet cells [13, 14], likely by counteracting the Wnt signaling pathway [15]. In addition, members of the epidermal growth factor (EGF) family of proteins likely serve as major drivers of TA cell proliferation through activation of the Ras-ERK pathway [8, 16].

Gut commensal bacteria are thought to constitute a key extrinsic factor in the homeostatic regulation of IECs [17]. Indeed, the proliferative activity or turnover rate of IECs is markedly reduced in germ-free mice [17] or in mice treated with a mixture of antibiotics [18], suggesting that commensal bacteria are important for the proliferation of these cells in the crypt. The mechanism by which commensal bacteria regulate homeostasis of the intestinal epithelium remains poorly understood, however. We have now examined which types of commensal bacteria are important for promotion of IEC turnover in the mouse small intestine, as well as which bioactive molecules produced by these bacteria underlie such regulation.

## Materials and Methods

### Ethics Statement

This study was approved by the Institutional Animal Care and Use Committee of Kobe University (Permit Number: P120304-R2, P120508-R2, P130206-R1, P150109) and Sankyo Labo Service Corporation, Inc (Permit Number: SKL-J-13049), and animal experiments were performed according to Animal Experimentation Regulations of Kobe University and Sankyo Labo Service Corporation, Inc. All efforts were made to minimize suffering.

### Mice

C57BL/6J mice were obtained from CLEA Japan (Tokyo, Japan) or Japan SLC (Hamamatsu, Japan). Myd88-deficient or Toll-like receptor 2-deficient mice were obtained from Oriental Bioservice, Inc. (Kyoto, Japan). These mice were maintained at the Institute for Experimental Animals at Kobe University Graduate School of Medicine under the specific pathogen–free (SPF) condition. Germ-free (GF) mice (C57BL/6N or C57BL/6J background) were obtained from Sankyo Labo Service (Tokyo, Japan) or CLEA Japan. GF mice or exGF mice, the latter of which had been treated orally with chloroform-treated feces of SPF mice, were maintained in vinyl isolators at Sankyo Labo Service after treatment. The health of mice was checked daily. Mice did not become severely ill or die at any time prior to the experimental endpoint. At the experimental endpoint, the mice were euthanized by carbon dioxide or were anesthetized with pentobarbital followed by cervical dislocation.

### Antibodies and reagents

A mouse monoclonal antibody (mAb) to β-catenin (# 610153) was obtained from BD Biosciences (San Diego, CA), a mouse mAb to β-tubulin (# T4026) was from Sigma-Aldrich (St. Louis, MO), a mouse mAb to cyclin D1 (# sc-450) was from Santa Cruz Biotechnology (Santa Cruz, CA), a mouse mAb to v-Src (# OP07) was from Merck Millipore (Billerica MA), and a rat mAb to bromodeoxyuridine (BrdU) (# ab6326) was from Abcam (Cambridge, MA). Rabbit polyclonal antibodies (pAbs) to Ki67 (# AM11168PU-S) were obtained from Acris (Herford, Germany), rabbit pAbs to mucin 2 (# sc-15334) were from Santa Cruz Biotechnology, rabbit pAbs to lysozyme (# A0099) were from Dako (Glostrup, Denmark), rabbit pAbs to ERK1 and ERK2 (ERK1/2) (# v1141) were from Promega (Madison, WI), and rabbit pAbs to cleaved-caspase3 (#9661S), to phosphorylated ERK1/2 (# 9101S), to AKT (# 9272S), to phosphorylated AKT (# 9271S), to S6 (# 2217S), to phosphorylated S6 (# 4858S), to STAT3 (signal transducer and activator of transcription 3) (# 9132S), to phosphorylated STAT3 (# 9145S), and to phosphorylated Src family kinases (p416Src) (# 2101S) were from Cell Signaling Technology (Beverly, MA). Secondary antibodies labeled with Cy3 or Alexa488 for immunofluorescence analysis were obtained from Jackson ImmunoResearch (West Grove, PA) and ThermoFisher (Waltham, MA), respectively, and horseradish peroxidase–conjugated goat secondary antibodies for immunoblot analysis were from Jackson ImmunoResearch. Ampicillin, vancomycin, metronidazole, sodium acetate, sodium butyrate, and sodium propionate were obtained from Wako (Osaka, Japan), and neomycin sulfate was from Nacalai Tesque (Kyoto, Japan). BrdU was from Sigma-Aldrich. Cyclopropanecarboxylic acid (CPC) was from Tokyo Chemical Industry (Tokyo, Japan). 4-chloro-α-(1-methylethyl)-N-2-thiazolylbenzeneacetamide (4-CMTB) was from Tocris Bioscience (Bristol, UK). U0126 was from Cell Signaling Technology.

### Depletion of gut commensal bacteria

Gut commensal bacteria were depleted as previously described [19]. In brief, 4-week-old mice were treated orally with an antibiotic cocktail (ampicillin, neomycin, and metronidazole each at 1 g/l, and vancomycin at 0.5 g/l) or with each antibiotic alone in drinking water for 4 weeks under the SPF condition.

### Administration of short-chain fatty acids (SCFAs) to vancomycin-treated mice

Mice at 4 weeks of age were treated orally with vancomycin (0.5 g/l) for 2 weeks and then with vancomycin (0.5 g/l) either alone or together with a mixture of SCFAs (62.5 mM acetate, 25.9 mM propionate, 40 mM butyrate) in drinking water for an additional 2 weeks under the SPF condition.

### Treatment of GF mice with chloroform-treated feces of SPF mice

Treatment with feces from SPF mice was performed as previously described [20] but with a slight modification. In brief, feces of adult SPF mice were suspended in 10 times volume (wt/vol) in phosphate-buffered saline (PBS), the suspension was filtered with a cell strainer (mesh size of 70 μm), and the filtrate was incubated with chloroform (final concentration of 3%) at 37°C for 1 h. Chloroform was then removed from the mixture by exposure to nitrogen gas. The chloroform-treated feces or chloroform-treated PBS as a control were administered to 8-week-old GF mice with the use of a gastric tube. Each mouse was then maintained individually in an isolator and analyzed at 3 weeks after treatment.

### Immunofluorescence analysis

The ileum was removed, fixed immediately for 3 h at room temperature with 4% paraformaldehyde in PBS, transferred to a series of sucrose solutions [7, 20, and 30% (wt/vol), sequentially] in PBS for cryoprotection, embedded in optical cutting temperature (OCT) compound (Sakura, Tokyo, Japan), and rapidly frozen in liquid nitrogen. Frozen sections with a thickness of 5 μm were prepared with a cryostat, mounted on glass slides, and air-dried. The sections were then subjected to immunofluorescence analysis with primary antibodies and fluorescent dye–labeled secondary antibodies as described previously [14, 21]. Images were acquired with a fluorescence microscope (BX51; Olympus, Tokyo, Japan).

### BrdU incorporation assay

Mice were injected intraperitoneally with BrdU (10 mg per kilogram of body weight). After 2, 48, 72, or 120 h, the ileum was removed and fixed with 4% paraformaldehyde, transferred to a series of sucrose solutions in PBS, embedded in OCT compound, and rapidly frozen with liquid nitrogen as described above for immunofluorescence analysis. Sections with a thickness of 5 μm were incubated for 30 min at 65°C with 0.025 M HCl, washed with 0.1 M borate buffer (pH 8.5), and incubated at room temperature first for 2 h with mAbs to BrdU and to β-catenin and then for 1 h with fluorescent dye–labeled secondary antibodies. Fluorescence images were obtained with a fluorescence microscope (BX51, Olympus). IEC migration distance was defined as the distance from the crypt base to the BrdU-positive cells that had migrated the farthest and was measured with the use of ImageJ software (NIH).

### Isolation of mouse IECs

Mouse IECs were isolated as previously described [22] but with a slight modification. In brief, the freshly isolated small intestine was washed with PBS, cut into small pieces, washed three times with Hanks’ balanced salt solution (HBSS) containing 1% fetal bovine serum and 25 mM HEPES-NaOH (pH 7.5), and then incubated three times on a rolling platform for 15 min at room temperature in HBSS containing 50 or 5 mM EDTA and 25 mM HEPES-NaOH (pH 7.5). After removal of tissue debris by centrifugation at 250 × *g* for 10 min at 4°C, IECs in the resulting supernatant were isolated by centrifugation at 250 × *g* for 10 min at 4°C and washed three times with PBS.

### Immunoblot analysis

Isolated IECs were lysed with radioimmunoprecipitation assay (RIPA) buffer [20 mM Tris-HCl (pH 7.5), 150 mM NaCl, 2 mM EDTA, 1% Nonidet P-40, 1% sodium deoxycholate, 0.1% sodium dodecyl sulfate, 50 mM NaF] containing 1 mM sodium vanadate and a protease inhibitor cocktail (Nacalai Tesque). The lysates were centrifuged at 17,500 × *g* for 15 min at 4°C, and the resulting supernatants were subjected to immunoblot analysis as previously described [21, 22].

### In situ hybridization

Expression of the olfactomedin4 (Olfm4) gene in the intestinal epithelium was examined by in situ hybridization performed as described previously [14]. In brief, paraffin-embedded sections of the ileum (thickness of 10 μm) were depleted of paraffin with xylene, rehydrated by exposure to a graded series of ethanol solutions, and treated with 0.2 M HCl and proteinase K. The sections were then fixed again with 4% paraformaldehyde and subjected first to demethylation by treatment with acetic anhydride and then to hybridization for 48 h at 65°C with a digoxigenin-labeled RNA probe for Olfm4 mRNA (IMAGE clone 1078130) at 500 ng/ml. They were then incubated overnight at 4°C with alkaline phosphatase–conjugated sheep pAbs to digoxigenin (Roche, Basel, Switzerland), washed, and incubated with nitroblue tetrazolium chloride and 5-bromo-4-chloro-3-indolyl phosphate (Sigma-Aldrich). Images were obtained with a fluorescence microscope (BX51, Olympus).

### Detection of 16S rRNA genes

Bacterial genomic DNA was isolated from fecal pellets with the use of a QIAamp DNA Stool Mini Kit (Qiagen, Hilden, Germany). Fragments of 16S rRNA genes were then amplified by PCR with the primers 5'-GGTGAATACGTTCCCGG-3' and 5'-TACGGCTACCTTGTTACGACTT-3' for total bacteria, 5'-CCTTCCGTGCCGSAGTTA-3' and 5'-GAATTAAACCACATACTCCACTGCTT-3' for *Clostridium leptum*, 5'-AAATGACGGTACCTGACTAA-3' and 5'-CTTTGAGTTTCATTCTTGCGAA-3' for *Clostridium coccoides*, 5'-GACGCTGAGGCATGAGAGCAT-3' and 5'-GACGGCACGGATTGTTATTCA-3' for segmented filamentous bacteria, and 5'-ATAGCCTTTCGAAAGRAAGAT-3' and 5'-CCAGTATCAACTGCAATTTTA-3' for *Bacteroides fragilis*.

### Measurement of SCFAs in cecal contents

Cecal contents were obtained from 8-week-old mice that had been treated orally (or not) with an antibiotic cocktail (ampicillin, neomycin, and metronidazole each at 1 g/l, and vancomycin at 0.5 g/l) for 4 weeks. The samples were diluted in sterile water, mixed with 2-ethylbutyrate as an internal control, and then labeled with 2-nitrophenylhydrazine with the use of a YMC-Pack FA kit (YMC, Kyoto, Japan). SCFAs in the samples were analyzed by liquid chromatography (Prominence LC-20AD; Shimadzu, Kyoto, Japan).

### Intestinal organoid culture

Intestinal organoid culture was performed as previously described [14, 16]. In brief, crypts were isolated from the small intestine by incubation for 30 min in PBS containing 2 mM EDTA. The isolated crypts were mixed with Matrigel (BD Biosciences) and transferred to 48-well plates. After polymerization of the Matrigel, advanced Dulbecco’s modified Eagle’s medium–F12 (Invitrogen) supplemented with penicillin-streptomycin (100 U/ml) (Invitrogen), 10 mM HEPES (Invitrogen), 1× GlutaMAX (Invitrogen), 1× N2 (Invitrogen), 1× B27 (Invitrogen), 1.25 mM *N*-acetylcysteine (Sigma-Aldrich), 10% R-spondin1–Fc–conditioned medium, and Noggin (100 ng/ml) (Peprotech) was overlaid on the gel in each well. The organoids were also treated (or not) with an SCFA cocktail (0.5 mM acetate, 0.5 mM propionate, 0.5 mM butyrate), each SCFA alone at 0.5 mM, 0.5 mM CPC, 5 μM 4-CMTB, or EGF (50 ng/ml) (Peprotech, Rocky Hill, NJ) and they were maintained in an incubator (37°C, 5% CO_2_) for 4 days. The organoids were then examined with a microscope (Axiovert 200; Carl Zeiss, Oberkochen, Germany), and the organoid area was measured by encircling the periphery of each organoid with the use of ImageJ software (NIH). For immunoblot analysis, organoids were cultured for 4 days and then incubated without EGF for 20 h before exposure to the SCFA cocktail or each SCFA alone for 20 min. Organoids were removed from the Matrigel with the use of Cell Recovery Solution (Corning, Corning, NY) and were lysed with RIPA buffer.

### Reverse transcription (RT) and real-time PCR analysis

Isolation of total RNA and quantitative RT-PCR analysis were performed as described previously [23], with minor modifications. In brief, total RNA was prepared from freshly isolated IECs with the use of Sepasol RNA I (Nacalai Tesque) and an RNeasy Mini Kit (Qiagen), and first-strand cDNA was synthesized from 0.8 μg of the RNA with the use of a QuantiTect Reverse Transcription Kit (Qiagen). The cDNA fragments of interest were amplified with the use of Fast Start SYBR Green Master (Roche, Penzberg, Germany) and a LightCycler 480 instrument (Roche). The amplification was analyzed with the use of LightCycler 480 software (Roche), and the abundance of each target mRNA was normalized by that of glyceraldehyde-3-phosphate dehydrogenase (GAPDH) mRNA. Primer sequences (forward and reverse, respectively) were as follows: GAPDH, 5'-AGGTCGGTGTGAACGGATTTG-3' and 5'-TGTAGACCATGTAGTTGAGGTCA-3'; c-Myc, 5'-CTGGATTTCCTTTGGGCGT-3' and 5'-TGGTGAAGTTCACGTTGAGGG-3'; Axin2, 5'-GGACTGGGGAGCCTAAAGGT-3' and 5'-AAGGAGGGACTCCATCTACGC-3'; Hes1, 5'-GGACAAACCAAAGACGGCCTCTGAGCACAG-3' and 5'-TGCCGGGAGCTATCTTTCTTAAGTGCATCC-3'; and Areg, 5'-TCTGCCATCATCCTCGCAGCTATT-3' and 5'-CGGTGTGGCTTGGCAATGATTCAA-3'.

### Statistical analysis

Data are presented as means ± standard error (SE) and were analyzed with Student’s *t* test or by analysis of variance (ANOVA) followed by Tukey’s test, as appropriate, with the use of GraphPad Prism software version 6.0 (GraphPad, San Diego, CA). A *P* value of <0.05 was considered statistically significant.

## Results

### Reduced proliferative activity and turnover of IECs in antibiotic-treated SPF mice

**Fig 1:**
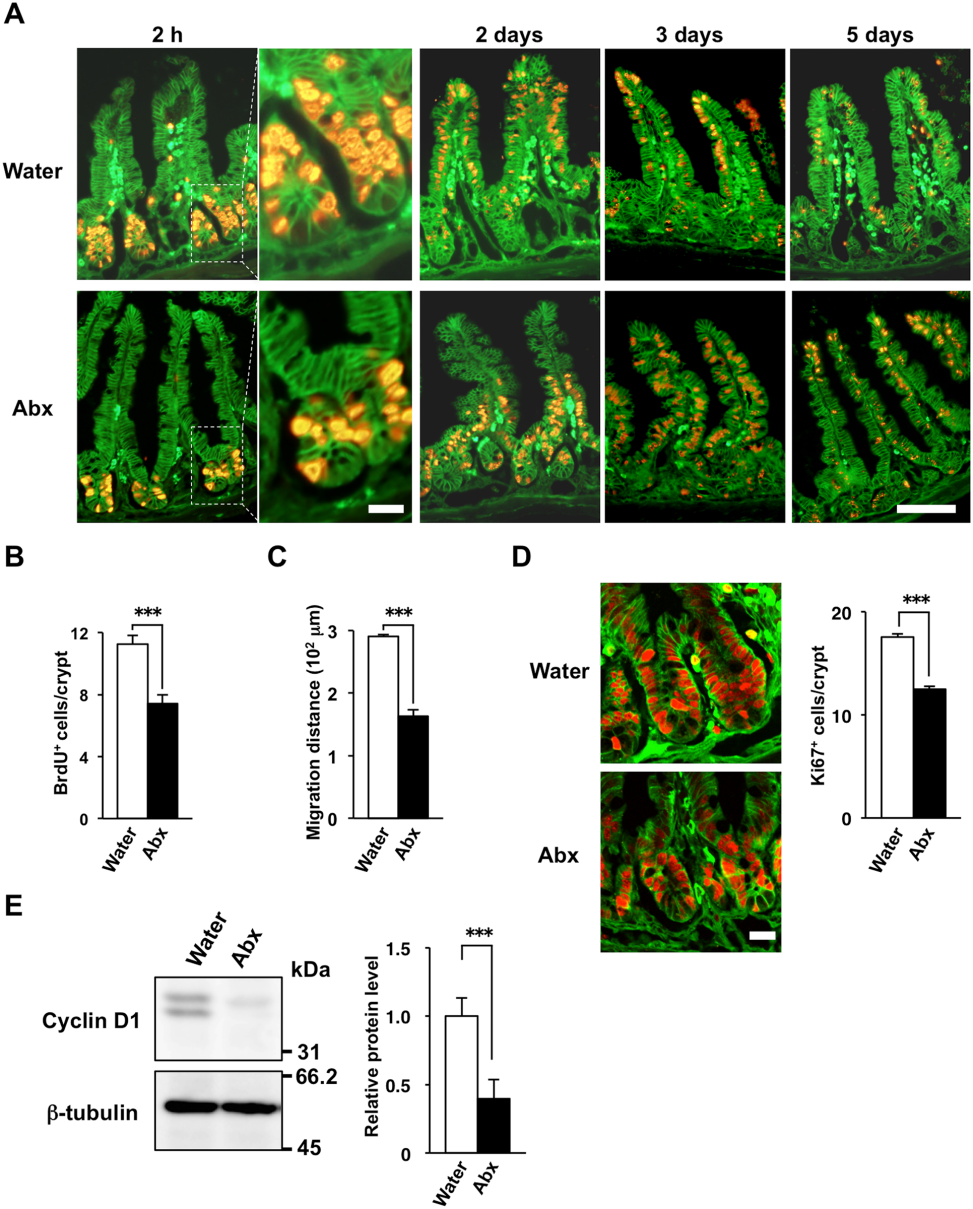
Reduced proliferative activity and turnover of IECs in antibiotic-treated SPF mice. (**A**) SPF mice (4-week-old) were provided with drinking water supplemented (or not) with an antibiotic cocktail (Abx: ampicillin, vancomycin, metronidazole, neomycin) for 4 weeks, after which frozen sections of the ileum from mice (8-week-old) were prepared at 2 h or 2, 3, or 5 days after BrdU injection and were immunostained with mAbs to BrdU (red) and to β-catenin (green). The boxed areas in the left panels for the sections prepared at 2 h after BrdU injection are shown at higher magnification in the right panels. Scale bars, 20 μm (higher magnification) or 100 μm (lower magnification). (**B**) The number of BrdU-positive cells per crypt at 2 h after BrdU injection was determined from sections similar to those in (A). Data are means ± SE for 30 crypts from one mouse and are representative of four mice. ***P < 0.001 (Student's *t* test). (**C**) The migration distance for BrdU-positive cells at 2 days after BrdU injection was determined from sections similar to those in (A). Data are means ± SE for 30 villi from one mouse and are representative of four mice. ***P < 0.001 (Student's *t* test). (**D**) Frozen sections prepared from the ileum of mice (8-week-old) treated (or not) with antibiotics as in (A) were immunostained with antibodies to Ki67 (red) and to β-catenin (green). Representative images are shown in the left panels. Scale bar, 20 μm. The number of Ki67-positive cells per crypt in such sections was also determined (right panel). Data are means ± SE for 30 crypts from one mouse and are representative of three mice. ***P < 0.001 (Student's *t* test). (**E**) IECs isolated from mice (8-week-old) treated (or not) with antibiotics as in (A) were lysed and subjected to immunoblot analysis with antibodies to cyclin D1 and to β-tubulin (loading control). Representative blots are shown in the left panels. The cyclin D1/β-tubulin band intensity ratio for such blots was also determined and is expressed relative to the value for control mice (right panel). Data are means ± SE from three individual experiments. ***P < 0.001 (Student’s *t* test).

To determine the turnover rate of IECs in the small intestine, we examined the incorporation of BrdU into these cells as well as their subsequent migration along the crypt-villus axis in SPF mice. At 2 h after a single injection of mice with BrdU, IECs in the crypts, most of which likely corresponded to TA cells, were labeled with BrdU (Fig 1A). The labeled cells had migrated up the villi at 2 to 3 days and most of them had disappeared at 5 days after BrdU injection (Fig 1A), indicating that the labeled IECs were released into the gut lumen after they had migrated to the tips of the villi. The continuous division of progenitor cells including TA cells in the crypt is thought to exert a force that acts on the existing mature IECs and promotes their migration along the crypt-villus axis [24–26], whereas cell loss from the villus tip is thought to give rise to negative pressure that also promotes IEC migration [25].

Depletion of commensal bacteria was previously shown to suppress the proliferative activity of crypt IECs [17, 18]. To reproduce this effect, we treated SPF mice with an antibiotic cocktail (ampicillin, vancomycin, metronidazole, neomycin) in drinking water for 4 weeks. Such treatment was previously shown to result in efficient depletion of commensal bacteria and a consequent marked reduction in the amounts of inflammatory cytokines such as interleukin-6 or tumor necrosis factor in the intestine [18, 19]. At 2 h after BrdU injection, the number of BrdU-positive IECs in crypts of the ileum was greatly reduced in the antibiotic-treated mice compared with control SPF mice (Fig 1A and 1B). In addition, the extent of migration of the BrdU-labeled IECs apparent at 2 days after BrdU injection was markedly reduced by the antibiotic treatment (Fig 1A and 1C). Indeed, a substantial number of labeled IECs was detected in the upper region of villi even at 5 days after BrdU injection in the antibiotic-treated mice (Fig 1A). Immunofluorescence staining for Ki67, a marker for cell proliferation [27], also showed a significant decrease in the number of Ki67-positive cells in crypts of the ileum in the antibiotic-treated mice compared with the control animals (Fig 1D). Moreover, immunoblot analysis revealed that the abundance of cyclin D1, another marker for cell proliferation [19], was markedly reduced in IECs isolated from the small intestine of antibiotic-treated mice (Fig 1E). Together, these results thus confirmed that treatment of mice with antibiotics results in suppression of the proliferative activity of IECs in the small intestine, as previously described [18]. In addition, we found that such treatment markedly slowed the migration of IECs along the crypt-villus axis, suggesting that the turnover of IECs is attenuated by the antibiotic treatment.

**Fig 2:**
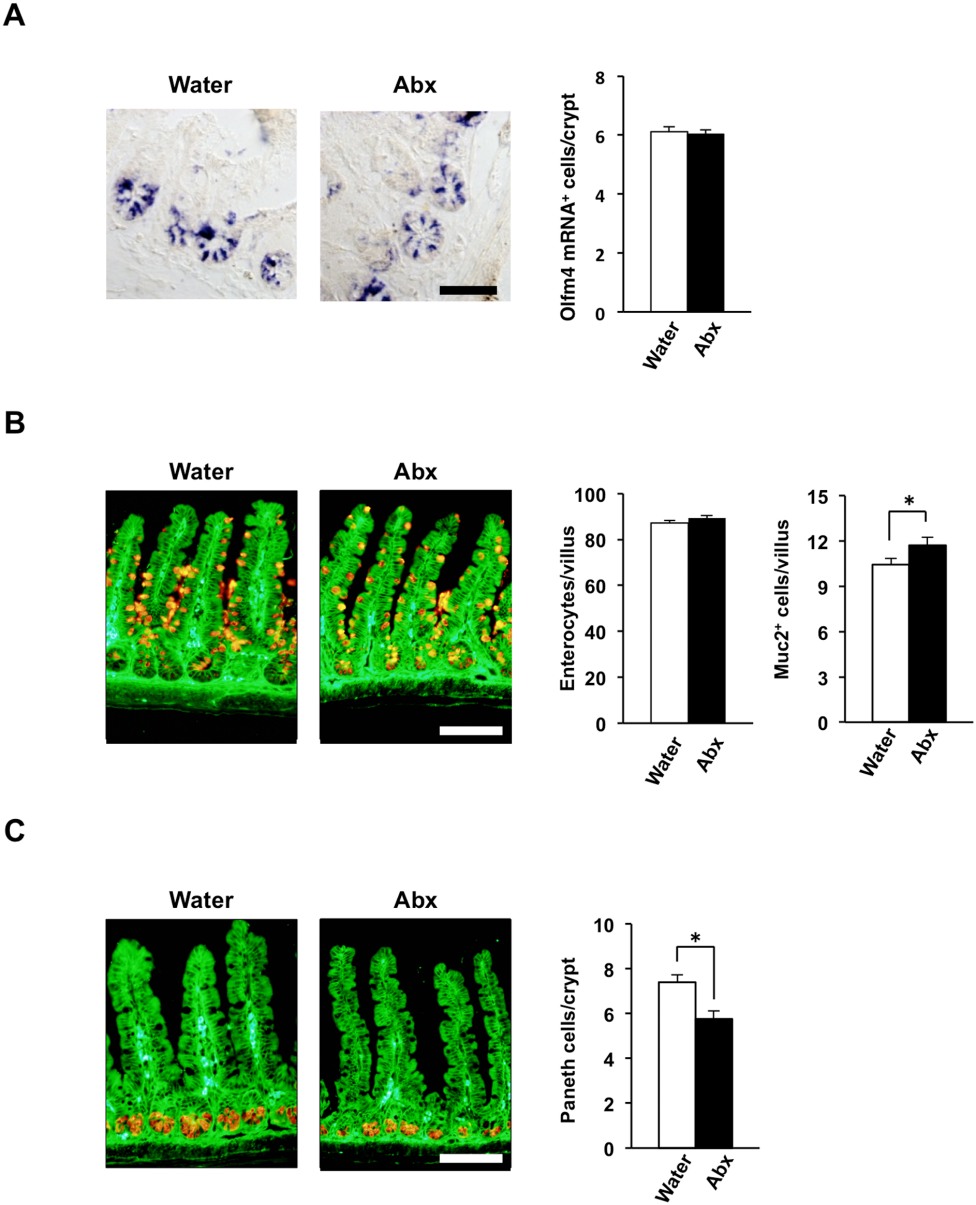
Numbers of different types of IECs in antibiotic-treated mice. (**A**) SPF mice (4-week-old) were provided with drinking water supplemented (or not) with an antibiotic cocktail (Abx: ampicillin, vancomycin, metronidazole, neomycin) for 4 weeks, after which paraffin-embedded sections of the ileum from mice (8-week-old) were prepared and subjected to in situ hybridization analysis of Olfm4 mRNA. Representative images are shown in the left panels. Scale bar, 50 μm. The number of Olfm4 mRNA–positive cells per crypt was also determined from such sections (right panel). Data are means ± SE for 25 crypts from one mouse and are representative of three mice. (**B**) Frozen sections prepared from the ileum of mice (8-week-old) treated as in (A) were immunostained with antibodies to Muc2 (red) and to β-catenin (green). Representative images are shown in the left panels. Scale bar, 100 μm. The numbers of β-catenin–positive absorptive enterocytes and Muc2-positive goblet cells per villus were also determined from such sections. Data are means ± SE for 30 villi from one mouse and are representative of seven mice. *P < 0.05 (Student’s *t* test). (**C**) Frozen sections prepared from the ileum of mice (8-week-old) treated as in (A) were immunostained with antibodies to lysozyme (red) and to β-catenin (green). Representative images are shown in the left panels. Scale bar, 100 μm. The number of lysozyme-positive Paneth cells per crypt was also determined from such sections (right panel). Data are means ± SE for 30 crypts from one mouse and are representative of seven mice. *P < 0.05 (Student’s *t* test).

We next examined whether the antibiotic treatment affected the numbers of different types of IECs in the intestinal epithelium. The number of Olfm4 mRNA–positive intestinal stem cells [28] in crypts of the ileum did not differ significantly between control and antibiotic-treated mice (Fig 2A). Moreover, the number of absorptive enterocytes in the ileum, which were identified on the basis of their morphology in tissue sections stained with a mAb to β-catenin [21], was not affected by antibiotic treatment (Fig 2B). The number of mucin 2 (Muc2)–positive goblet cells was slightly increased (Fig 2B), whereas that of lysozyme-positive Paneth cells was slightly reduced, in the ileum of antibiotic-treated mice compared with the control mice (Fig 2C).

### Importance of Gram-positive commensal bacteria for the proliferative activity and turnover of IECs

**Fig 3:**
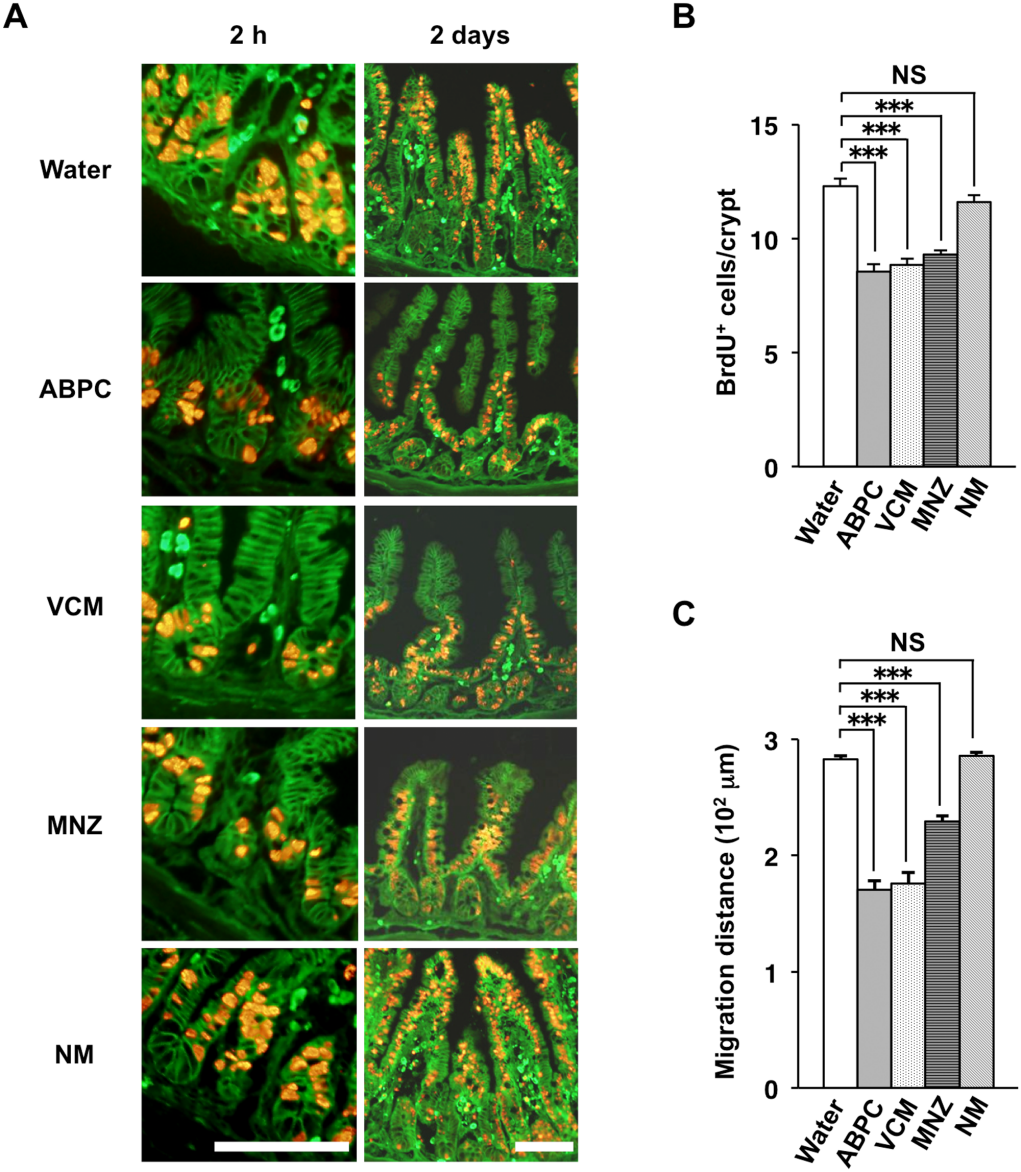
Importance of Gram-positive commensal bacteria for the proliferative activity and turnover of IECs. (**A**) SPF mice (4-week-old) were provided with drinking water supplemented (or not) with ampicillin (ABPC), vancomycin (VCM), metronidazole (MNZ), or neomycin (NM) for 4 weeks, after which frozen sections of the ileum from mice (8-week-old) were prepared at 2 h or 2 days after BrdU injection and were immunostained with mAbs to BrdU (red) and to β-catenin (green). Scale bars, 100 μm. **(B)** The number of BrdU-positive cells per crypt at 2 h after BrdU injection was determined from sections similar to those in (A). Data are means ± SE for 30 crypts from one mouse and are representative of three mice. ***P < 0.001 (ANOVA and Tukey’s test); NS, not significant. (**C**) The migration distance for BrdU-positive cells at 2 days after BrdU injection was determined from sections similar to those in (A). Data are means ± SE for 30 villi from one mouse and are representative of four mice. ***P < 0.001 (ANOVA and Tukey’s test).

We next examined which of the antibiotics in the cocktail are responsible for suppression of the proliferative activity of crypt IECs in the ileum. SPF mice were treated with ampicillin, vancomycin, metronidazole, or neomycin individually in drinking water for 4 weeks. The incorporation of BrdU into crypt IECs at 2 h after BrdU injection was attenuated by treatment with ampicillin, vancomycin, or metronidazole, but not by that with neomycin (Fig 3A and 3B). The migration of BrdU-positive IECs along the crypt-villus axis was also inhibited in mice treated with either ampicillin, vancomycin, or metronidazole, whereas it was unaffected in those treated with neomycin (Fig 3A and 3C). The former three antibiotics are effective against Gram-positive bacteria, whereas neomycin preferentially targets Gram-negative bacteria [29, 30]. These data thus suggested that Gram-positive bacteria in the intestine promote both the proliferation and turnover of IECs in the small intestine.

### Promotion of the proliferative activity and turnover of IECs in GF mice by chloroform-resistant commensal bacteria

**Fig 4:**
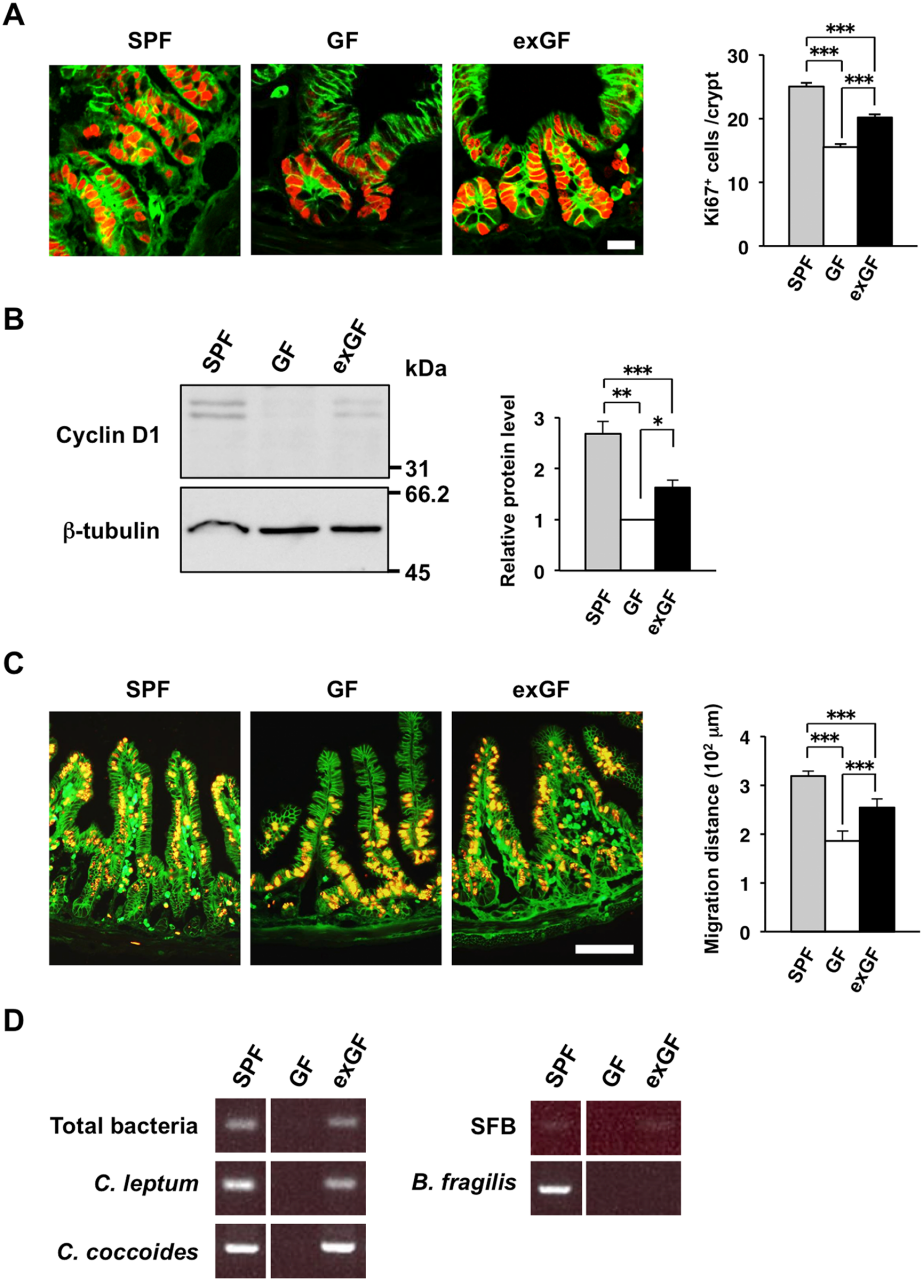
Promotion of the proliferative activity and turnover of IECs in GF mice by chloroform-resistant commensal bacteria. (**A**) Chloroform-treated feces from untreated SPF mice or chloroform-treated PBS as a control were administered orally to 8-week-old GF mice. The resulting exGF and control mice, respectively, were housed separately for 3 weeks in isolators, after which frozen sections of the ileum from both groups of mice (11-week-old) as well as from age-matched SPF mice were immunostained with antibodies to Ki67 (red) and to β-catenin (green). Representative images are shown in the left panels. Scale bar, 20 μm. The number of Ki67-positive cells per crypt was also determined from such sections (right panel). Data are means ± SE for 30 crypts from one mouse and are representative of four mice. ***P < 0.001 (ANOVA and Tukey’s test). (**B**) Lysates of IECs isolated from mice (11-week-old) treated as in (A) were subjected to immunoblot analysis with antibodies to cyclin D1 and to β-tubulin (left panels). The cyclin D1/β-tubulin band intensity ratio was also determined from such blots and expressed relative to the value for GF mice (right panel). Data are means ± SE from three separate experiments. *P < 0.05, **P < 0.01, ***P < 0.001 (ANOVA and Tukey’s test). (**C**) Frozen sections of the ileum were prepared from SPF, GF, and exGF mice (11-week-old) at 2 days after BrdU injection and were immunostained with mAbs to BrdU (red) and to β-catenin (green). Representative images are shown in the left panels. Scale bar, 100 μm. The migration distance for BrdU-positive cells was also determined from such sections (right panel). Data are means ± SE for 30 villi from one mouse and are representative of three mice. ***P < 0.001 (ANOVA and Tukey’s test). (**D**) PCR analysis of 16S rRNA genes of total bacteria, *C*. *leptum*, *C*. *coccoides*, segmented filamentous bacteria (SFB), or *B*. *fragilis* in fecal pellets from GF, exGF, or SPF mice (11-week-old).

As in antibiotic-treated mice, the proliferative activity of IECs in crypts of germ-free (GF) mice was previously shown to be attenuated compared with that in SPF mice [17, 31]. We also found that the number of Ki67-positive IECs in crypts as well as the abundance of cyclin D1 in isolated IECs were markedly reduced for GF mice compared with SPF mice (Fig 4A and 4B). In addition, the migration of BrdU-labeled IECs was slowed in GF mice compared with SPF mice (Fig 4C). Given that our results suggested that Gram-positive bacteria are important for promotion of the proliferative activity of crypt IECs, we next examined the effects of administration of chloroform-treated feces of SPF mice to GF mice. Treatment of feces with chloroform is thought to result in enrichment of spore-forming Gram-positive bacteria such as *Clostridium* species [32]. GF mice subjected to oral administration of chloroform-treated feces (exGF mice) or of chloroform-treated PBS as a control were housed separately for 3 weeks in isolators. The number of Ki67-positive IECs in crypts as well as the abundance of cyclin D1 in isolated IECs were significantly increased in exGF mice compared with control GF mice (Fig 4A and 4B). The migration distance of BrdU-positive IECs along the crypt-villus axis was also greater in exGF mice compared with control GF mice (Fig 4C). These results thus suggested that chloroform-resistant bacteria, such as *Clostridium* species, play an important role in promotion of both the proliferative activity and turnover of IECs in the small intestine. Indeed, PCR analysis of bacterial 16S rRNA genes revealed that *Clostridium* species (*C*. *leptum* and *C*. *coccoides*) and segmented filamentous bacteria, but not the Gram-negative bacterium *Bacteroides fragilis*, were present in the feces of exGF mice (Fig 4D).

### Promotion of the proliferative activity and turnover of IECs by SCFAs

**Fig 5:**
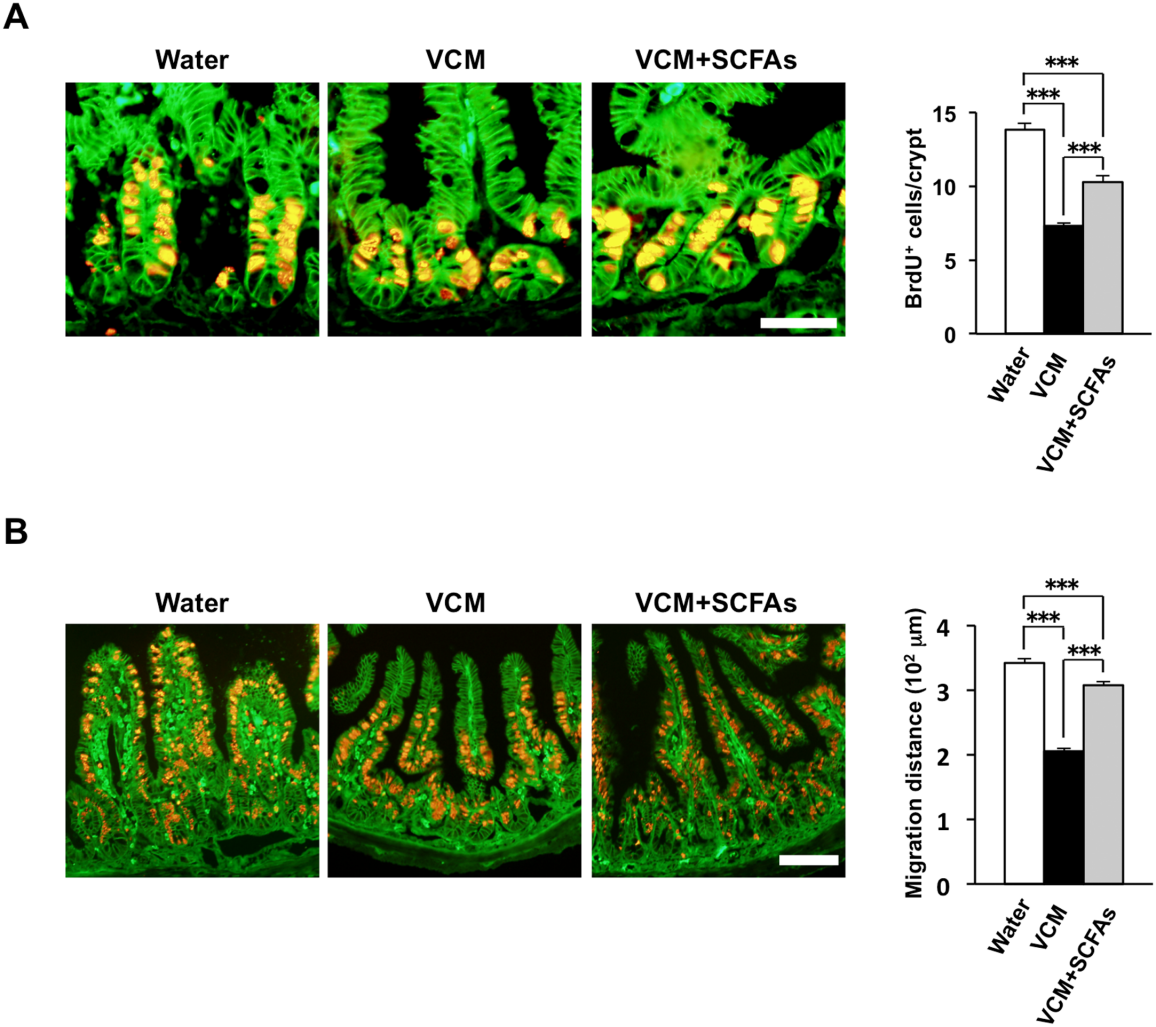
Promotion of the proliferative activity and turnover of IECs by SCFAs. (**A**) SPF mice (4-week-old) were provided with drinking water supplemented (or not) with vancomycin (VCM) for 2 weeks and then with vancomycin alone or together with an SCFA cocktail (62.5 mM acetate, 25.9 mM propionate, 40 mM butyrate) for an additional 2 weeks. The mice (8-week-old) were then injected with BrdU, and frozen sections of the ileum prepared at 2 h after the injection were immunostained with mAbs to BrdU (red) and to β-catenin (green). Representative images are shown in the left panels. Scale bar, 100 μm. The number of BrdU-positive cells per crypt was also determined from such sections (right panel). Data are means ± SE for 30 crypts from one mouse and are representative of three mice. ***P < 0.001 (ANOVA and Tukey’s test). (**B**) Frozen sections of the ileum from mice (8-week-old) treated as in (A) were also prepared at 2 days after BrdU injection and subjected to immunostaining as in (A). Representative images are shown in the left panels. Scale bar, 100 μm. The migration distance for BrdU-positive cells was also determined from such sections (right panel). Data are means ± SE for 30 villi from one mouse and are representative of three mice. ***P < 0.001 (ANOVA and Tukey’s test).

SCFAs, such as acetate, propionate, and butyrate, are bacterial fermentation products, and their abundance was previously shown to be reduced in the cecal or colonic contents of antibiotic-treated mice or GF mice [33, 34]. We confirmed that the amounts of acetate, propionate, and butyrate were all markedly reduced in the cecal contents of SPF mice treated with the antibiotic cocktail (S1 Table). Given that chloroform-resistant spore-forming Gram-positive bacteria, such as *Clostridium* species, are thought to be largely responsible for the production of SCFAs in the intestine [20, 35, 36], we next examined the effects of SCFAs on the proliferative activity of crypt IECs as well as on IEC migration in SPF mice treated with vancomycin. Administration of a mixture of SCFAs (acetate, propionate, butyrate) in drinking water significantly increased the number of BrdU-labeled IECs in crypts (Fig 5A) as well as promoted the migration of BrdU-positive IECs along the crypt-villus axis (Fig 5B) in vancomycin-treated mice.

**Fig 6:**
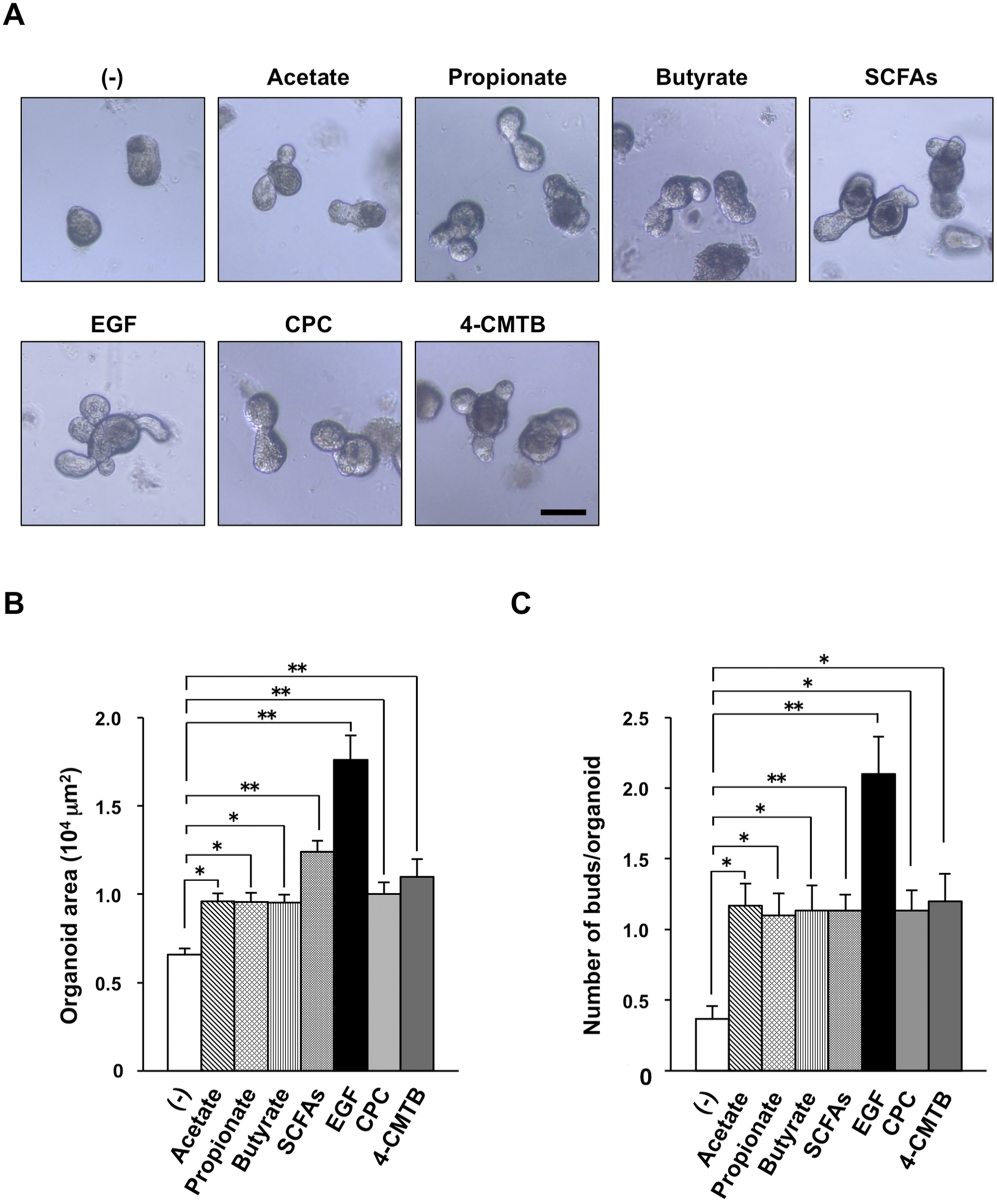
Promotion of the development of intestinal organoids by SCFAs. (**A**) Mouse intestinal organoids were incubated in the absence (–) or presence of either 0.5 mM acetate, 0.5 mM propionate, 0.5 mM butyrate, SCFAs (mixture of 0.5 mM acetate, 0.5 mM propionate, and 0.5 mM butyrate), 50 mg/ml EGF, 0.5 mM CPC (GPR41 agonist), or 5 μM 4-CMTB (GPR43 agonist) for 4 days. The organoids were then examined by light microscopy. Scale bar, 100 μm. (**B** and **C**) Organoid area (B) and the number of buds per organoid (C) determined for cultures treated as in (A). Data are means ± SE from three separate experiments, with 30 organoids being examined for each condition in each experiment. *P < 0.05, **P < 0.01 (ANOVA and Tukey’s test).

To clarify whether the promotion of IEC proliferation by SCFAs in vivo is attributable to a direct action of SCFAs on IECs, we next examined the effects of SCFAs on the development of mouse intestinal organoids in vitro. As shown previously [16], EGF markedly promoted organoid development (Fig 6A) as determined by measurement of both organoid size (Fig 6B) and budding (Fig 6C). Acetate, propionate, and butyrate also each significantly promoted organoid development, and the effect of a mixture of all three SCFAs was greater than those of the individual fatty acids (Fig 6). These results thus suggested that the promotion of IEC proliferation and turnover by SCFAs in vivo is due, at least in part, to a direct effect of SCFAs on these cells. SCFAs are known as ligands for G-protein coupled receptors (GPRs) such as GPR41 and GPR43 [37, 38]. We found that either a GPR41 agonist, CPC, or a GPR43 agonist, 4-CMTB, promoted organoid development (Fig 6). Therefore, either GPR41 or GPR43 is likely important for the promotion by SCFAs of IEC proliferation and turnover.

### Reduced phosphorylation levels of ERK, S6, and STAT3 in the small intestine of antibiotic-treated SPF mice

**Fig 7:**
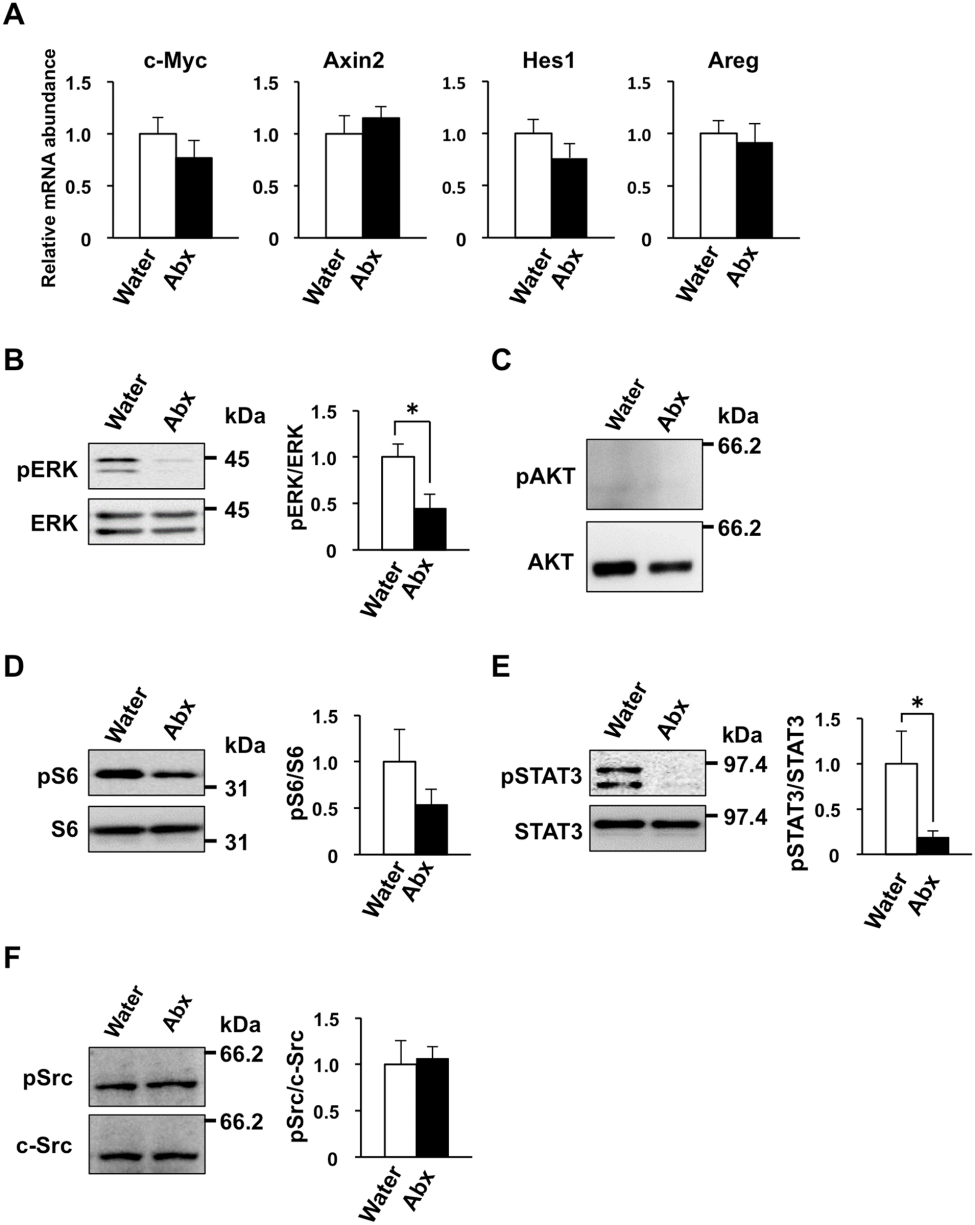
Reduced phosphorylation levels of ERK, S6, and STAT3 in the small intestine of antibiotic-treated SPF mice. (**A**) Mice (4-week-old) were provided with drinking water supplemented (or not) with an antibiotic cocktail (Abx: ampicillin, vancomycin, metronidazole, neomycin) for 4 weeks, after which the abundance of mRNAs for c-Myc, Axin2, Hes1, and Areg in freshly isolated IECs from mice (8-week-old) was determined by quantitative RT-PCR analysis. The amount of each mRNA was normalized by that of GAPDH mRNA and is expressed relative to the corresponding value for control mice. Data are means ± SE from four mice. (**B**–**F**) Lysates of IECs prepared from mice (8-week-old) treated as in (A) were subjected to immunoblot analysis with antibodies to total or phosphorylated (p) forms of ERK (B), AKT (C), S6 (D), STAT3 (E), or c-Src (F). Representative blots and densitometric analysis of the ratio of the band intensity for the phosphorylated protein to that for the total protein are shown, with the quantitative data being expressed relative to the corresponding value for control mice and presented as means ± SE from three separate experiments. *P < 0.05 (Student’s *t* test).

We next examined whether the activity of signaling pathways thought to be important for IEC proliferation [8, 16] is affected by treatment of mice with antibiotics. We first determined the abundance of mRNAs for genes targeted by the Wnt (c-Myc, Axin2) or Notch (Hes1) signaling pathways in IECs isolated from SPF mice treated with the antibiotic cocktail for 4 weeks. Quantitative RT-PCR analysis revealed that the amounts of c-Myc, Axin2, and Hes1 mRNAs did not differ significantly between IECs from control or antibiotic-treated mice (Fig 7A). The transcriptional co-activator YAP/TAZ, whose abundance is negatively regulated by the Hippo signaling pathway [39], was recently shown to contribute to the promotion of IEC proliferation [40]. However, the abundance of mRNA derived from the YAP target gene for amphiregulin (Areg) also did not differ between IECs from control or antibiotic-treated mice (Fig 7A).

**Fig 8:**
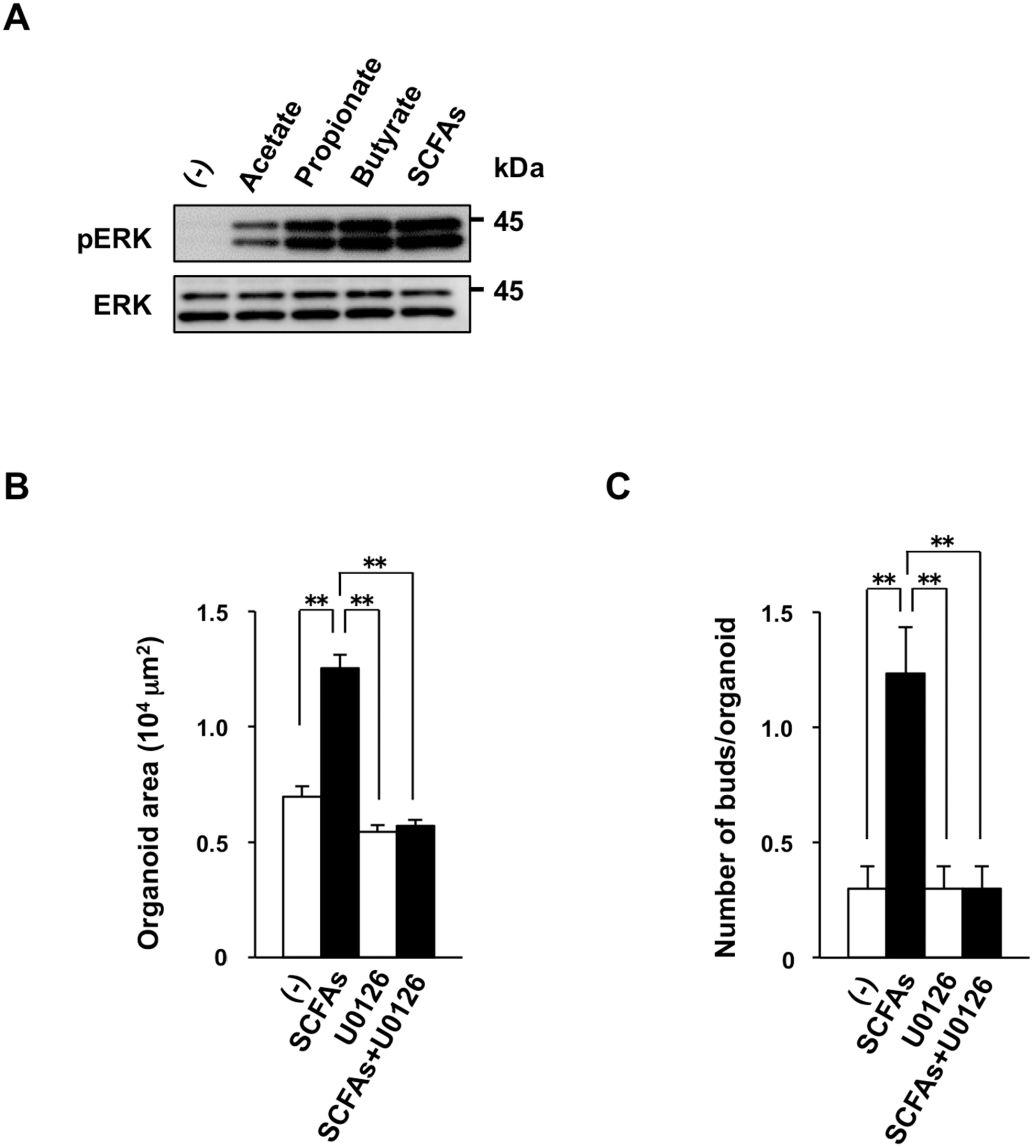
Importance of MEK-ERK signaling in the SCFA-dependent development of intestinal organoids. (**A**) Mouse intestinal organoids were stimulated (or not) with 0.5 mM acetate, 0.5 mM propionate, 0.5 mM butyrate, or SCFAs (mixture of 0.5 mM acetate, 0.5 mM propionate, and 0.5 mM butyrate) for 20 min, after which they were lysed and subjected to immunoblot analysis of phosphorylated and total forms of ERK. (**B** and **C**) Mouse intestinal organoids were incubated in the absence (–) or presence of SCFAs (mixture of 0.5 mM acetate, 0.5 mM propionate, and 0.5 mM butyrate) with or without MEK inhibitor (10 μM U0126) for 4 days. Organoid area (B) and the number of buds per organoid (C) determined for cultures. Data are means ± SE from three separate experiments, with 30 organoids being examined for each condition in each experiment. **P < 0.01 (ANOVA and Tukey’s test).

The phosphorylation level of ERK, a signaling molecule thought to be important for IEC proliferation [16], was markedly reduced in IECs from antibiotic-treated mice compared with those from control mice (Fig 7B). The AKT–mTORC1 (mammalian target of rapamycin complex 1)–S6 signaling pathway is also thought to be important for IEC proliferation [41, 42]. The phosphorylated form of AKT was not detected in IECs from control or antibiotic-treated mice (Fig 7C), whereas the amount of phosphorylated ribosomal protein S6 tended to be reduced in IECs from antibiotic-treated mice compared with those from control mice (Fig 7D). Both STAT3 [43] and Src family kinases [40] are also implicated in the proliferation of IECs. The amount of phosphorylated STAT3 was markedly reduced in IECs from antibiotic-treated mice (Fig 7E), whereas the amount of phosphorylated c-Src did not differ between IECs from antibiotic-treated or control mice (Fig 7F). Finally, we also found that acetate, propionate, butyrate, or a mixture of all three SCFAs markedly increased the phosphorylation of ERK in intestinal organoids (Fig 8A). In contrast, a MEK inhibitor, U0126, inhibited the SCFA-promoted development of intestinal organoids (Fig 8B and 8C). These data suggest that the MEK-ERK signaling is important for SCFA-induced development of intestinal organoids.

## Discussion

We have here shown that the proliferative activity of crypt IECs as well as the migration of mature IECs along the crypt-villus axis are markedly attenuated in both antibiotic- treated SPF mice and GF mice. In contrast, antibiotic treatment did not significantly affect the numbers of Olfm4 mRNA–positive stem cells or absorptive enterocytes, and it induced only a small increase or decrease in the numbers of goblet cells and Paneth cells, respectively. These observations thus indicate that commensal bacteria stimulate the proliferation of crypt IECs, most likely TA cells, and then promote the migration and turnover of mature IECs, whereas they are not important for the differentiation of the individual mature cell types from their progenitors. We also found that antibiotics selective for Gram-positive bacteria were most effective at inhibiting the proliferation of crypt IECs as well as the migration of mature IECs. Furthermore, treatment of GF mice with chloroform-treated feces of SPF mice, which are enriched in spore-forming Gram-positive bacteria, resulted in a marked increase in both the proliferative activity of crypt IECs and the migration of mature IECs. Gram-positive commensal bacteria, such as *Clostridium* species, are thus likely important for the proliferation of cryptic IECs and IEC turnover.

The mechanism by which Gram-positive commensal bacteria regulate IEC turnover remains to be fully elucidated. They might directly or indirectly promote IEC turnover by activation of a Toll-like receptor (TLR)–mediated signaling pathway. Gram-positive bacteria are thought to activate TLR2 through their peptidoglycan or lipoprotein membrane components [44]. However, the proliferation of colonic epithelial cells is increased by the loss of Myd88, which is required for signal transduction by TLR2 [19]. Indeed, we found that the number of BrdU-labeled IECs in crypts was increased rather than reduced in mice deficient in TLR2 or Myd88 compared with control SPF mice (S1 Fig). In addition, the abundance of cyclin D1 in IECs isolated from these two mutant strains of mice was also increased compared with that in those from wild-type mice (S1 Fig). It therefore appears unlikely that Gram-positive bacteria promote IEC turnover through activation of a TLR2-mediated signaling pathway. We also found that the amount of cleaved-caspase 3 protein, an apoptosis marker, was markedly reduced in IECs of antibiotic-treated SPF mice (S2 Fig), suggesting that commensal bacteria promoted the turnover of, as well as apoptosis of, IECs.

Gram-positive bacteria, such as *Clostridium* species, are thought to contribute to the production of SCFAs by fermentation of dietary fiber [45]. We have now shown that oral administration of a mixture of SCFAs restored the proliferative activity of crypt IECs as well as the migratory activity of mature IECs in vancomycin-treated SPF mice. Moreover, either acetate, butyrate, or propionate alone or a mixture of all three SCFAs markedly promoted the development of intestinal organoids in vitro. These observations thus indicate that SCFAs are important for promotion of the proliferation and turnover of IECs by Gram-positive commensal bacteria.

To investigate further the molecular mechanism by which commensal bacteria regulate IEC turnover, we examined the activation state of various signaling pathways that are thought to be important for IEC proliferation. The expression of Wnt or Notch target genes was unchanged in IECs of antibiotic-treated mice. This finding is consistent with our observations that the numbers of stem cells or Paneth cells, the development or homeostasis of which largely depends on Wnt signaling [8], as well as the number of goblet cells, the generation of which depends on the Notch pathway [46], were not substantially affected by antibiotic treatment. In contrast, we found that phosphorylation of ERK was markedly attenuated in IECs of antibiotic-treated mice. Moreover, SCFAs induced the phosphorylation of ERK in intestinal organoids. Inhibition of MEK-ERK signaling prevented the SCFA-dependent development of intestinal organoids. SCFAs are known to activate ERK signaling through GPRs such as GPR41 and GPR43 [38]. We indeed found that both a GPR41 agonist and a GPR43 agonist promoted the development of intestinal organoids. Collectively, these data suggest that promotion of the proliferative activity and turnover of IECs by commensal bacteria is mediated by SCFA-induced activation of ERK signaling. In addition, activation by SCFAs of GPR41 or GPR43 may participate in such role of commensal bacteria.

In addition to ERK signaling [8, 16], the mTORC1-S6 pathway and gp130-STAT3 pathway are thought to contribute to regulation of the proliferation of IECs or their progenitors [41, 43]. Indeed, the phosphorylation levels of S6 and STAT3 were reduced in IECs of antibiotic-treated mice. One or both of these signaling pathways may thus also be important for promotion of IEC turnover by commensal bacteria.

In summary, our results indicate that Gram-positive commensal bacteria are a major determinant of the turnover of mature IECs, with their stimulatory action likely being mediated through the production of SCFAs. Further investigation will be required to clarify whether activation of ERK, S6, or STAT3 signaling is indeed important for the promotion of IEC turnover by commensal bacteria. Moreover, the molecular mechanisms by which commensal bacteria activate these three signaling pathways remain to be determined.

## Supporting Information

S1 Fig Promotion of the proliferative activity of IECs in Myd88 KO or TLR2 KO mice. (**A**) Eight-week-old wild-type (WT), Myd88 KO or TLR2 KO mice were injected with BrdU, and frozen sections of the ileum prepared at 2 h after the injection were immunostained with mAbs to BrdU (red) and to β-catenin (green). Representative images are shown in the left panels. Scale bar, 100 μm. The number of BrdU-positive cells per crypt was also determined from such sections (right panel). Data are means ± SE for 30 crypts. **P < 0. 01, ***P < 0.001 (ANOVA and Tukey’s test). (**B**) Frozen sections of the ileum from mice were also prepared at 2 days after BrdU injection and subjected to immunostaining as in (A). Representative images are shown in the left panels. Scale bar, 100 μm. The migration distance for BrdU-positive cells was also determined from such sections (right panel). Data are means ± SE for 30 villi. NS, not significant. (**C**) IECs isolated from mice were lysed and subjected to immunoblot analysis with antibodies to cyclin D1 and to β-tubulin (loading control). Blots are shown in the left panels. The cyclin D1/β-tubulin band intensity ratio for such blots was also determined and is expressed relative to the value for WT mice (right panel).
(TIF)

S2 Fig Reduction of the amount of cleaved-caspase3 in IECs from antibiotic-treated SPF mice. Mice (4-week-old) were provided with drinking water supplemented (or not) with an antibiotic cocktail (Abx: ampicillin, vancomycin, metronidazole, neomycin) for 4 weeks, after which lysates of IECs prepared from mice (8-week-old) were subjected to immunoblot analysis with antibodies to cleaved-caspase3 and to β-tubulin (loading control). Representative blots are shown in the left panels. The cleaved-caspase3/β-tubulin band intensity ratio for such blots was also determined and is expressed relative to the value for control mice (right panel). Data are means ± SE from three individual experiments. *P < 0.05 (Student’s *t* test).
(TIF)

S1 Table Amounts of acetate, propionate, and butyrate in cecal contents of SPF mice treated with an antibiotic cocktail (Abx: ampicillin, vancomycin, metronidazole, neomycin) in drinking water for 4 weeks. (PDF)
