# Supplementary figures and images for: Promotion of Intestinal Epithelial Cell Turnover by Commensal Bacteria: Role of Short-Chain Fatty Acids
Source: PLoS One. 2016 May 27;11(5):e0156334. doi: 10.1371/journal.pone.0156334 (PMC4883796; doi:10.1371/journal.pone.0156334)

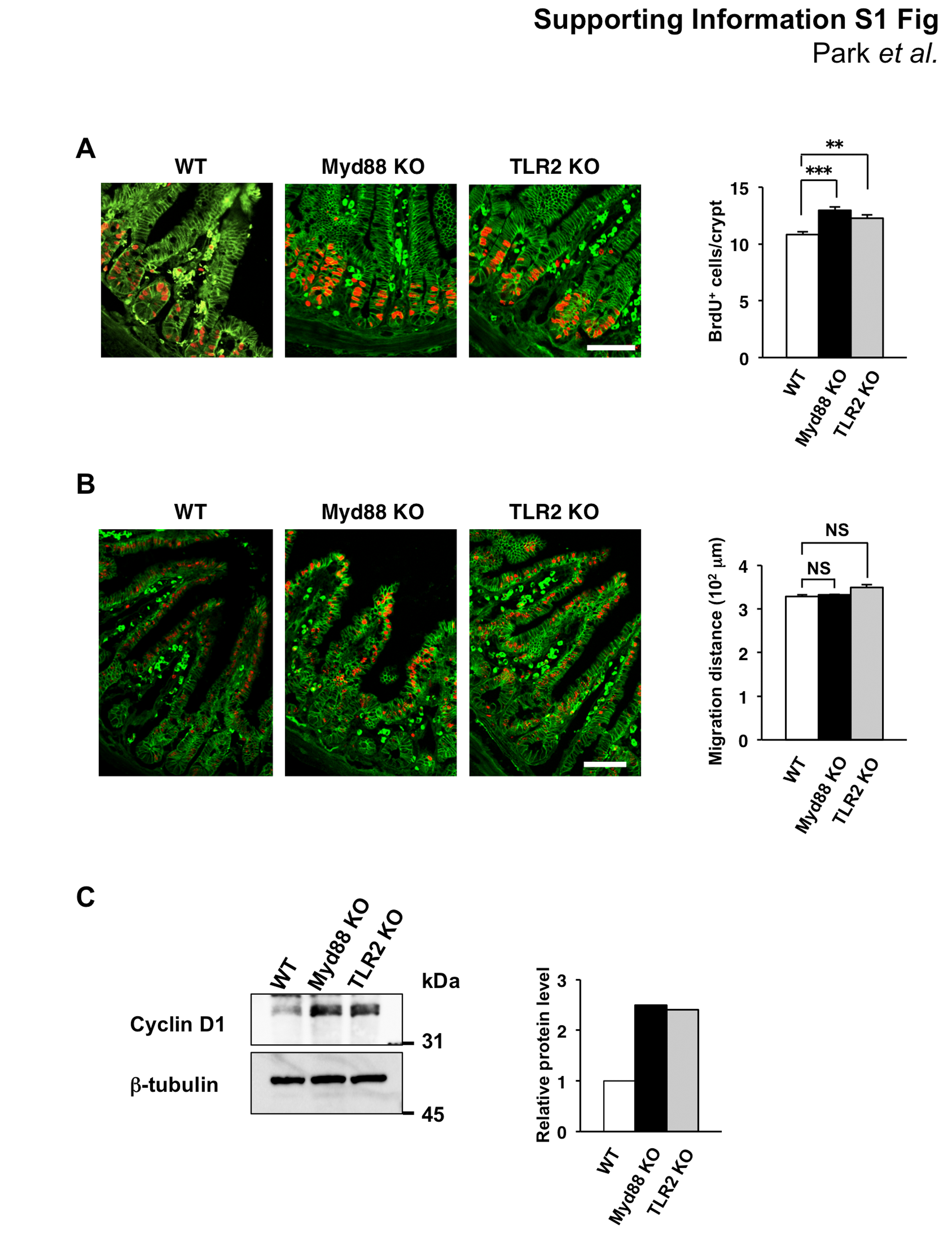

Supplement: S1 Fig — (A) Eight-week-old wild-type (WT), Myd88 KO or TLR2 KO mice were injected with BrdU, and frozen sections of the ileum prepared at 2 h after the injection were immunostained with mAbs to BrdU (red) and to β-catenin (green). Representative images are shown in the left panels. Scale bar, 100 μm. The number of BrdU-positive cells per crypt was also determined from such sections (right panel). Data are means ± SE for 30 crypts. **P < 0. 01, ***P < 0.001 (ANOVA and Tukey’s test). (B) Frozen sections of the ileum from mice were also prepared at 2 days after BrdU injection and subjected to immunostaining as in (A). Representative images are shown in the left panels. Scale bar, 100 μm. The migration distance for BrdU-positive cells was also determined from such sections (right panel). Data are means ± SE for 30 villi. NS, not significant. (C) IECs isolated from mice were lysed and subjected to immunoblot analysis with antibodies to cyclin D1 and to β-tubulin (loading control). Blots are shown in the left panels. The cyclin D1/β-tubulin band intensity ratio for such blots was also determined and is expressed relative to the value for WT mice (right panel). (TIF) [file pone.0156334.s001.tif]

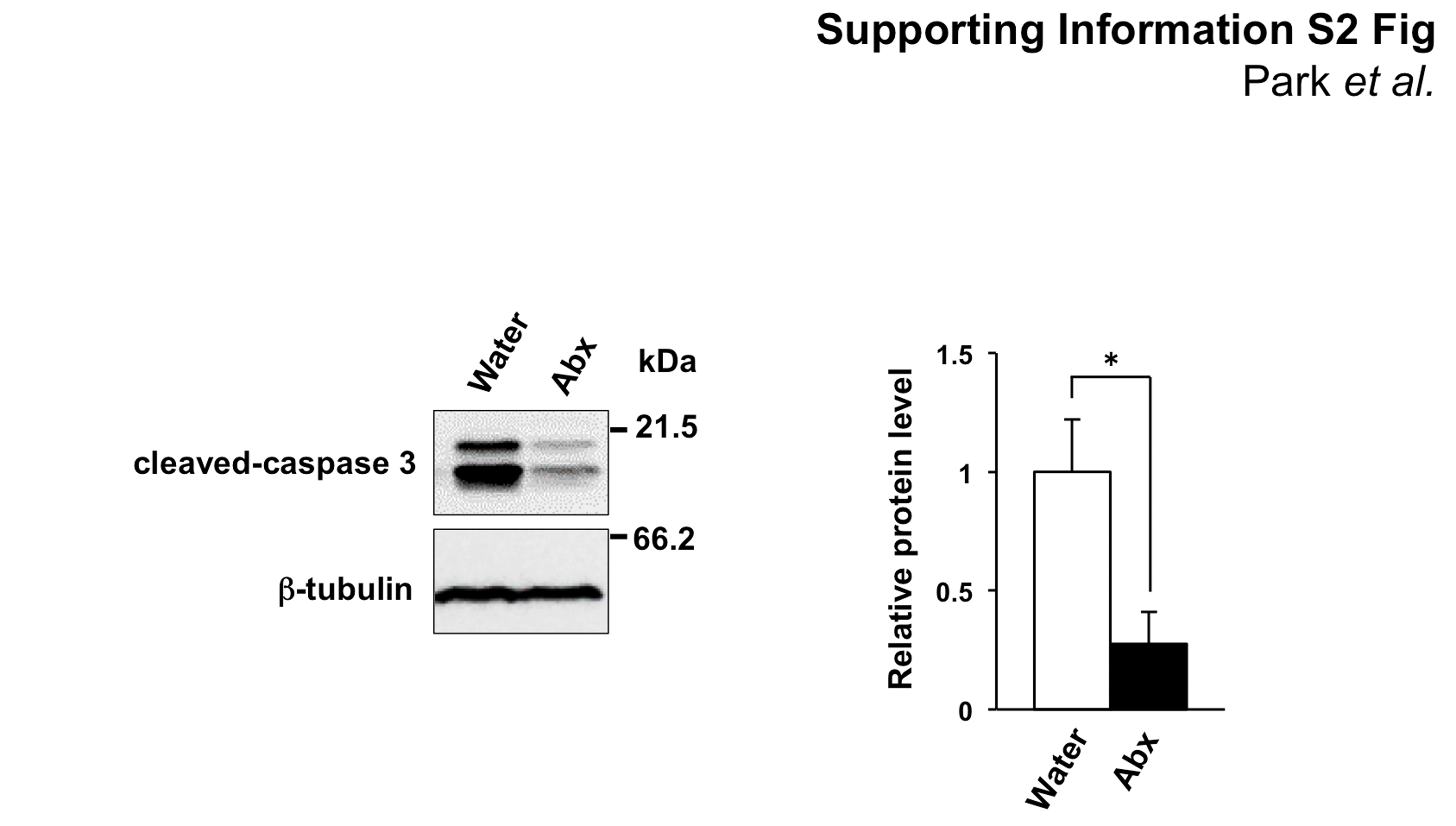

Supplement: S2 Fig — Mice (4-week-old) were provided with drinking water supplemented (or not) with an antibiotic cocktail (Abx: ampicillin, vancomycin, metronidazole, neomycin) for 4 weeks, after which lysates of IECs prepared from mice (8-week-old) were subjected to immunoblot analysis with antibodies to cleaved-caspase3 and to β-tubulin (loading control). Representative blots are shown in the left panels. The cleaved-caspase3/β-tubulin band intensity ratio for such blots was also determined and is expressed relative to the value for control mice (right panel). Data are means ± SE from three individual experiments. *P < 0.05 (Student’s t test). (TIF) [file pone.0156334.s002.tif]
