# Supplementary material for: Promotion of Intestinal Epithelial Cell Turnover by Commensal Bacteria: Role of Short-Chain Fatty Acids
Source: PLoS One. 2016 May 27;11(5):e0156334. doi: 10.1371/journal.pone.0156334 (PMC4883796; doi:10.1371/journal.pone.0156334)
Supplement: S1 Table — (PDF) [file pone.0156334.s003.pdf]

S1 Table 1. Amounts of acetate, propionate, and butyrate in cecal contents of SPF mice treated with an antibiotic cocktail (Abx: ampicillin, vancomycin, metronidazole, neomycin) in drinking water for 4 weeks.

SCFA (nmol/mg cecal contents)	Water	Abx
Acetate	31.87	3.72
Propionate	7.02	ND
Butyrate	13.45	ND

ND, not detected
